# Epigenetic relay: Polycomb-directed DNA methylation in mammalian development

**DOI:** 10.1371/journal.pgen.1011854

**Published:** 2025-09-15

**Authors:** Teresa Urli, Maxim V. C. Greenberg

**Affiliations:** Université Paris Cité, CNRS, Institut Jacques Monod, Paris, France; University of Washington, UNITED STATES OF AMERICA

## Abstract

In mammals, repression of germline-specific gene expression is essential for preserving somatic cell identity and preventing disease. Germline gene silencing is often dependent on the presence of promoter 5-cytosine DNA methylation (5meC). Early mammalian development, however, is marked by a dramatic loss of 5meC levels genome-wide. Recent research has highlighted a specialized variant of the Polycomb Repressive Complex 1, PRC1.6, as a key regulator that maintains transient silencing of germline genes in this embryonic window. Eventually, PRC1.6 seems to stimulate the recruitment of *de novo* DNA methyltransferases (DNMTs), although the precise mechanisms remain to be fully elucidated. Evidence suggests a coordinated epigenetic relay, potentially involving direct protein interactions and shaping the local histone modification landscape. This review explores PRC1.6 as a central epigenetic hub that connects Polycomb repression, histone 3 lysine 9 (H3K9) methylation, and DNA methylation pathways. Unraveling this interplay will provide key insights into the mechanisms that maintain the critical barrier between the germline and the soma, essential not only for proper development but also for preserving somatic cell function and health throughout life.

## Introduction

Epigenetic pathways, involving both DNA methylation (5-methylcytosine, 5meC) and histone post-translational modifications (PTMs), are key regulators of chromatin structure and gene expression, and are essential for establishing and maintaining cell identity [1]. Among these, 5meC represents a particularly stable repressive pathway. This modification is crucial not only for the silencing of repetitive sequences, such as transposable elements (TEs), but also for the long-term somatic inactivation of specific classes of protein-coding genes [2]. Genes exhibiting high density of 5meC at their promoters broadly fall into three main groups: imprinted genes, genes on the inactive X chromosome in female cells, and germline-specific genes [3]—sometimes subcategorized as cancer-testis antigen genes (CATs) [4] and/or germline “genome-defense” genes (GGDs) [5]. It should be noted that there is also evidence for tissue-specific methylation signatures in a small proportion of high CpG-dense promoters [6].

Promoter regions of many protein-coding genes overlap with CpG-rich sequences, known as CpG islands (CGIs). While most CGIs remain DNA methylation-free in mammals, 5meC provides a durable silencing mechanism when enriched at these sequences [7,8]. However, the early stages of mammalian development are accompanied by extensive reprogramming of methyl-cytosine. This entails a genome-wide erasure of 5meC that occurs after fertilization, followed by a rapid reestablishment of the mark through the activity of the *de novo* DNA methyltransferases, DNMT3A and 3B, that is concomitant with the restriction of pluripotency potential [9,10]. This phenomenon raises important questions about how the three categories of genes with promoter high density 5meC—imprinted genes, X-linked genes, and germline-specific genes—navigate this reprogramming phase, while the vast majority of other CGIs are protected from deposition of the methyl-mark.

Imprinted control regions (ICRs) arrive DNA-methylated from the gametes in a parental allele-specific manner. An important feature of ICRs is their ability to withstand embryonic 5meC erasure. The mechanism behind this protection generally relies on the binding of the KRAB-containing zinc finger (KRAB-ZF) factors, ZFP57 and ZFP445, which recognize methylated motifs and recruit co-repressors, including DNMTs [11,12].

X-linked genes undergo distinct 5meC dynamics. When 5meC levels are at their lowest, in the naïve pluripotent inner mass cells of the blastocyst—also known as the “ground state”—both X chromosomes are active. Random X chromosome inactivation (XCI) is initiated during the exit of the naïve period, where the long non-coding RNA *Xist* recruits a number of chromatin regulators, including a specific set of Polycomb group (PcG) proteins [13]. PcG proteins fall into two Polycomb Repressive Complexes (PRCs): PRC1, which monoubiquitinates histone H2A at lysine 119 (H2AK119ub), and PRC2, which trimethylates histone H3 at lysine 27 (H3K27me3) [14]. 5meC deposition at CGIs along the inactive X (Xi) occurs relatively late during XCI, contributing to long-term, stable repression [15,16]. At least for a subset of X-linked CGIs, 5meC gain depends on the protein SMCHD1, which in turn is stabilized and recruited to the Xi by PRC1-H2AK119ub [16,17]. Thus, in the context of XCI, cooperation between PcG and DNA methylation allows for a tightly locked repressive state.

Finally, in the ground state, many germline gene promoters are also initially silenced in a 5meC-independent manner through the PcG pathway. Interestingly, Polycomb repression and DNA methylation are generally found to be mutually exclusive at CGIs, suggesting a regulatory antagonism between these two pathways [18–20]. This interplay becomes especially significant during early embryogenesis, when most Polycomb-regulated CGI promoters resist *de novo* 5meC deposition [21]. Notable exceptions include inactive X-linked genes and germline genes, the latter of which are the focus of this review [7,8].

**Fig 1:**
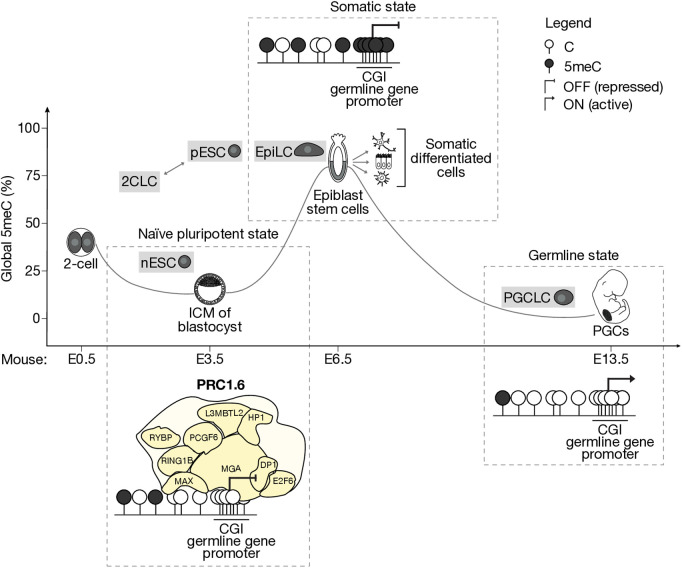
Epigenetic transition from PRC1.6-mediated repression to DNA methylation (5meC) ensures tight regulation of germline genes and supports the establishment of the germline–soma barrier. In the naïve ground state of the blastocyst, the genome is globally hypomethylated (low 5meC), and in the pluripotent cells of the inner cell mass (ICM, in gray), a group of germline genes is repressed via the activity of PRC1.6, which binds their CpG-rich promoters (CpG island, CGI, promoter). 5meC deposition at these sites occurs during implantation, leading to stable silencing of these genes in epiblast stem cells (gray) that persists in somatic lineages. During germline specification, this repression is lifted as 5meC is erased, reaching its lowest levels in primordial germ cells (PGCs). These cells are situated in the embryo’s genital ridges at embryonic day 13.5 (E13.5). These regulatory dynamics can be recapitulated *in vitro* by the cellular types highlighted in gray. 2CLC, 2-cell-like cell; nESC, naïve embryonic stem cells; pESC, primed ESC (serum-grown); EpiLC, epiblast-like cell; PGCLC, PGC-like cell. Corresponding mouse embryonic days are reported along the x-axis.

A specialized form of Polycomb repression via the PRC1 subcomplex, PRC1.6, has emerged as a key player in the PcG-5meC transition at germline genes [22,23] (Fig 1). PRC1.6 harbors DNA-binding subunits that target germline gene promoters and appears to act upstream of 5meC, potentially facilitating its deposition at these sites [22,23]. Importantly, during germline specification, genome-wide 5meC demethylation [10] contributes to the reactivation of this same cohort of genes, including those required for meiosis and gametogenesis [24–26].

Overall, the expression of germline genes must be tightly regulated both spatially (germline vs soma) and temporally. This necessity is underscored by their aberrant activation in cancers [27,28], as well as by the onset of fertility defects when they are prematurely derepressed in the germline [26]. These findings likely account for the convergence of multiple epigenetic pathways to ensure robust control of this gene class.

In this review, we focus on the unique features of PRC1.6 and its role as a precursor of 5meC, which ultimately enforces the boundary between the somatic and germline transcriptional programs. We begin by summarizing the discoveries that established PRC1.6 as a key repressor of germline genes. We then explore emerging insights into how this complex interacts with DNA methylation machinery, potentially acting as a molecular bridge between Polycomb-mediated repression and long-term gene silencing. Finally, we conclude by discussing the biological consequences of PRC1.6 dysfunction, particularly its implications in cancer, where inappropriate activation of germline genes is frequently observed.

## PRC1.6 composition

The first description of PRC1.6 dates back to the early 2000s, in a study characterizing the repressive transcription factor E2F6 [29]. Accordingly, PRC1.6 was initially referred to as E2F6.com (E2F6 complex). Immunoprecipitation (IP) of E2F6 followed by mass spectrometry revealed PRC1 proteins RING1A/B, PCGF6 (also known as MBLR), and YAF2 (a RYBP homolog). In addition to E2F6 and its heterodimeric binding partner DP1, the DNA-binding factors Myc-associated factor X (MAX) and MAX gene-associated protein (MGA) also interact with E2F6, indicating the potential for targeting multiple sequence motifs. Furthermore, L3MBTL2 and other subunits linked with histone 3 lysine 9 (H3K9) methylation, such as G9a (also known as EHMT2) and GLP (EHMT1), and the reader protein HP1γ (CBX3) were detected in association with E2F6. Subsequent proteomics approaches led to the refinement of PRC1.6 composition and the inclusion of additional subunits, such as RYBP, WDR5, HDAC1, HDAC2, and HP1β (CBX1) [30–33]. Notably, the exact complex composition may vary depending on many factors, such as at the organismal level (human or mouse), tissue level (cellular states), and genome level (target genes). Recently, proximity-based labeling has expanded the known PRC1.6 interactome [34], identifying factors such as KDM5C, ATF7IP, SETDB1, and RIF1, which will all be discussed in this review. These findings provide valuable resources for investigating PRC1.6 crosstalk with other epigenetic pathways.

Classically, PRC1 contains specific chromodomain proteins (CBX2, 4, 6–8) that target it to chromatin by binding to H3K27me3, thus reinforcing canonical Polycomb-mediated silencing. In contrast, PRC1.6 is one of the mammalian “variant PRC1s (vPRC1s)”, which lack these canonical PRC1 members and can nucleate on chromatin independently of PRC2 [35]. Below, we provide a detailed description of our current understanding of the PRC1.6 functional subunits, which are also summarized in Table 1.

**Table 1:** PRC1.6 subunits and selected interactors. List of PRC1.6 components subdivided by class (PcG, DNA-binding, H3K9 methylation, Other). “Core” components, as defined by [30] are highlighted in yellow. PRC1.6 interactors mentioned in this review are also listed.

PcG	
RING1A or B	PRC1 catalytic core component, catalyzes H2AK119ub. Direct interaction with PCGF6 is essential for its recruitment at PRC1.6 targets.
RYBP or YAF2	Binds H2AK119ub, stimulates RING1A/B catalytic activity.
PCGF6	PRC1.6 characterizing factor, contributes to the integrity of the complex.
**DNA-binding**	
E2F6/DP1	Heterodimer that binds E2F motif; contributes to targeting of PRC1.6.
MAX/MGA	Heterodimer that binds E-box and (MGA) T-box motifs; contributes to targeting of PRC1.6. MGA also exhibits scaffolding properties, supporting stability of the complex.
**H3K9 methylation**	
G9A/GLP	Function as heterodimer to catalyze H3K9me1/2.
HP1γ (and or β)	Recognize and bind H3K9me2/3 through their chromodomain.
SETDB1	Catalyzes H3K9me3.
ATF7IP	SETDB1 partner.
**Other**	
L3MBTL2	Supports binding to some PRC1.6 targets.
HDAC1/2	Histone deacetylases.
WDR5	Member of H3K4 methyltransferase complexes; uncharacterized function within PRC1.6.
USP7	Deubiquitinating enzyme, promotes PRC1.6 stability. Independently implicated in 5meC maintenance.
ZMYM2	SUMO binding protein, possibly supporting PRC1.6-dependent 5meC gain.
KDM5C	H3K4me2/3 demethylase.
RIF1	Stabilizes PRC1.6 facilitating its binding at some loci. Independently discovered as a regulator of ERVs.

### Polycomb-related factors

RING1A (also known as RING1) and its homolog RING1B (RING2 or RNF2) are part of the core of both canonical and variant PRC1 complexes, and they are the catalytic subunits that deposit H2AK119ub [36]. In mice, loss of RING1A/B results in severe embryonic lethality [37–39]. Furthermore, H2AK119ub is a platform for binding by the PRC2 subunit JARID2 [40], reinforcing PRC1/PRC2 cooperative activity. However, the specific role of RING1A/B in PRC1.6 may be more nuanced. A study in mouse embryonic stem cells (ESCs) showed that PRC1.6 is often associated with active genes, low H2AK119ub, and sometimes no RING1A/B [41]. As will be discussed, the predominant silencing role of PRC1.6 may rely on other complex components [32,42]. It is worth noting that some other results argue for an important role of RING1B catalytic activity in the repression of at least some targeted genes (*Ddx4*, *Mael*, *Tdrd1*, *Piwil2*) [33].

The RYBP/YAF2 subunit contains an Npl4 zinc finger (NZF) domain that binds H2AK119ub and promotes the recruitment of variant PRC1 complexes, including PRC1.6, to previously marked chromatin sites [43,44]. This stimulates the spreading of H2AK119ub through a positive-feedback loop [44]. Reduction of *Rybp* expression in mouse ESCs results in failure to differentiate to embryoid bodies and is correlated with lower cell proliferation and loss of H2AK119ub [30]. Pertinently, a role of this protein in silencing germline genes was reported in mouse ESCs [45]. Remarkably, forced targeting of RYBP could efficiently inhibit the transcription of a reporter gene, and this repression was maintained after the release of ectopic tethering. This was accompanied by a gradual loss of H2AK119ub, but a gain in 5meC at the previously targeted promoter [44].

PCGF6 is one of six PCGF proteins, each of which is the characterizing factor of its respective PRC1 subcomplex [30,41]. *Pcgf6* knock-out (KO) causes pleiotropic defects in both placenta and embryo development. Moreover, *Pcgf6*−/− mice are not born at the normal Mendelian ratio, and lethality can be detected throughout pre- and post-implantation [33]. Surviving *Pcgf6*-depleted mice show fertility defects and ectopic expression of germ cell-related genes (further validated in this study: *Ddx4*, *Luzp4*, *Mael*, *Syce1*, *Slc25a31*, *Rbpm46*, *Tex11*, and *Xlr4c*) in somatic tissues, such as the brain and liver [42]. Even if germline gene dysregulation was the predominant transcriptional signature of the *Pcgf6* KO model, *Hox* gene expression alteration and correlated homeotic transformations were also reported [33]. This could pose interpretative challenges in regards to the observed phenotypes, which could be linked to germline gene derepression, developmental gene dysregulation, or both.

At the molecular level, this factor is essential for the integrity of the PRC1.6 complex, as its absence leads to missing subunits in co-IP experiments and is correlated with their reduced chromatin binding [42,46]. There is substantial overlap between RING1B- and PCGF6-bound sites in mouse ESCs, and such loci frequently overlap promoters of germline-specific genes. PCGF6 plays a critical role in recruiting RING1B to these sites [33,42,47], and this recruitment depends on their physical interaction [33]. Importantly, loss of either PCGF6 or RING1A/B leads to derepression of germline genes.

Despite this, some PCGF6 targets (*Mael*, *Syce1*, *Slc25a31*, *Ddx4*, *Tdrkh*, and *Tcam1*) showed only a marginal decrease in H2AK119ub levels in *Pcgf6* KO ESCs, even if these genes were upregulated and RING1B binding was strongly impacted. Instead, decreased H3K9me1/2 and elevated acetylation of H3 and H4 were observed, suggesting PCGF6 may recruit histone methyltransferases and deacetylases not generally associated with PRC1 activity [42]. Supporting this hypothesis, reduced binding of G9A/GLP and HDAC1/2 was detected at the same targets in *Pcgf6* KO ESCs [42].

Interestingly, RNA sequencing (RNA-seq) analysis revealed that a similar set of genes is derepressed upon *Pcgf6* deletion in naïve ESCs, epiblast-like cells (EpiLCs), and primordial germ cell-like cells (PGCLCs) (see Box 1 for description of these cell types); however, different phenotypic outcomes were reported in the three conditions. In detail, ablation of *Pcgf6* in ESCs showed growth arrest accompanied by cell death, while in EpiLCs, only growth defects were reported. In contrast, a positive impact on differentiation and proliferation was detected if the depletion was induced in PGCLCs [33]. These results highlight the importance of PCGF6/PRC1 activity in the naïve state, when DNA methylation levels are at their lowest point. Notably, interpretation from *in vitro* data should be considered with caution, as culture artifacts could confound results. For example, EpiLCs are hypermethylated compared to epiblast stem cells, which becomes exacerbated in extended culture [21]. Additional studies in mouse ESCs also reported a role for PCGF6 in self-renewal capacity [46,48], and this was correlated with the regulation of genes implicated in differentiation and spermatogenesis [46].

### DNA-binding factors

A distinguishing feature of PRC1.6 compared to typical mammalian Polycomb complexes is the presence of factors that bind to specific DNA motifs, typically enriched at gene regulatory elements. E2F6 is a repressive transcription factor (TF) part of the E2F family that has been implicated in the regulation of cell cycle and cell fate [49–51]. *E2f6* deletion does not impair mouse viability; however, it causes homeotic transformations, aligning with Polycomb function [52]. The E2F6/DP1 heterodimer recognizes the E2F-motif 5’-TCCCGC-3’ and contributes to PRC1.6 genome localization [23,53]. The role of E2F6 in germline gene repression was discovered quite early in studies on somatic cells [54,55]. The presence of the E2F6-motif in numerous members of this subclass of genes prompted further research in this direction [56].

Interestingly, E2F6 emerged as a necessary factor for the establishment of 5meC at promoters, dependent on DNMT3B activity [57]. This finding confirmed previous observations that also linked E2F6 repressive activity to CpG hypermethylation [55]. Comparison between E2F6 ChIP-seq and RNA-seq data in wild-type versus *E2f6* KO ESCs and mouse embryos confirmed the role of this protein as a repressor of a subset of germline genes [23]. The majority of these contain an E2F6-motif, in some cases accompanied by a MAX/MGA-recognized E-box motif.

MAX is a transcription factor containing a basic helix-loop-helix leucine zipper domain (bHLH-Zip). Initially identified as a binding partner of the proto-oncogene MYC, MAX is essential for MYC’s oncogenic activity [58–60]. Myc/MAX binds to the E-box motif (5′-CACGTG-3′) and recruits co-activators, such as histone acetyltransferases (HATs) and chromatin remodelers, promoting expression of genes associated with cell proliferation [61]. MAX also dimerizes with other bHLH-Zip proteins, such as members of the MAD/MDX and MNT families, acting in this case as a repressor, through the recruitment of histone deacetylases (HDACs) complexes [61,62].

In PRC1.6, MAX partners with MGA, which contains not only a bHLH-Zip domain but also a T-domain, another highly conserved DNA-binding motif [63]. The MAX/MGA heterodimer can thus bind to both E- and T-boxes and has a crucial role in PRC1.6 targeting [33,53,64]. Remarkably, deletion of MGA in a human cell line (HEK293) resulted in complete ablation of PRC1.6 binding (determined by colocalization of L3MBTL2, MGA, and PCGF6) [53]. This effect was only partially attributable to MGA’s DNA-binding activity, as the protein also functions as a scaffold within the complex [53]. Furthermore, *MGA* KO in HEK293 cells was accompanied by reduced E2F6 and PCGF6 protein levels detected by immunoblot, while transcript levels were unchanged. Conversely, in the absence of either L3MBTL2, E2F6, or PCGF6, protein levels of MGA and other PRC1.6 components were not altered [53]. In other words, MGA plays an outsized role in PRC1.6 activity that extends beyond its targeting ability.

MAX and MGA play a crucial role in early development, and their absence results in embryonic lethality. In mice, ablation of *Mga* is deleterious for the naïve pluripotent cells of the inner cell mass (ICM) and causes death of ESCs *in vitro* [65]. Concordantly, a previous RNA interference (RNAi) screen found MGA to be an essential factor for ESC self-renewal [48]. *Max* KO leads to generalized developmental arrest at early post-implantation mouse embryos (embryonic day 5.5–6.5, E5.5–E6.5), with a timing that reflects the exhaustion of maternal protein storage [66]. *Max* KO mouse ESCs, grown in classical serum conditions (i.e., high 5meC levels), fail to sustain the undifferentiated state and subsequently undergo extensive cell death. It should be noted that mutations in *Max* also lead to defective Myc/MAX activity, thus confounding its precise role within PRC1.6 [67].

The first link between MAX and germline gene regulation was made via an RNAi screen performed in mouse ESCs. While this study focused on *Max*, notably, few other hits were discovered as important for germline gene silencing, including two other PRC1.6 members—*L3mbtl2* and *Mga—*and the complex interactor *Atf7ip* [68]. In the same study, MAX-dependent repression of germline genes was shown to rely, at least in part, on G9A/GLP-mediated H3K9me2 deposition [68].

In a follow-up study, full ablation of *Max* in mouse ESCs resulted in a meiosis-like cytological phenotype, strengthening the evidence that MAX is a crucial regulator of germline gene expression. Moreover, this role was mechanistically linked to PRC1.6, as knockdowns (KDs) of different components of the complex led to the upregulation of a similar set of genes, while this was not the case for *Myc* and *Mad* families [69]. Consistently, *Max* and *Mga* KDs in mouse ESCs led to upregulation of a subset of germline genes bound concomitantly by MGA/MAX, PCGF6, and RING1B but not MYC [33].

### H3K9 methylation activity

**Fig 2:**
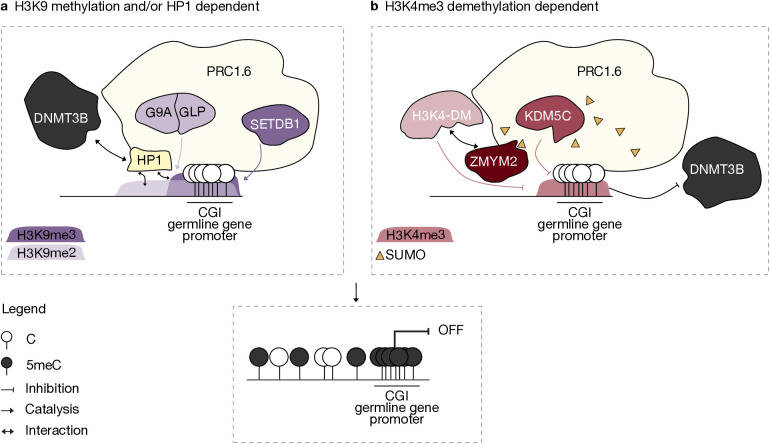
Two proposed models for PRC1.6-facilitated recruitment of 5meC at germline gene promoters. **a.** HP1-mediated recruitment model: PRC1.6 facilitates the deposition of H3K9me2/3 via recruitment or coordination with the histone methyltransferases G9A/GLP and SETDB1. These repressive marks are recognized by HP1 proteins, which are also PRC1.6 components and can interact with DNMT3B, thereby promoting localized *de novo* DNA methylation. **b.** H3K4 demethylation model: PRC1.6 promotes the removal of H3K4me3, a histone mark that blocks DNMT activity, through recruitment of KDM5C or other H3K4 demethylases (H3K4-DM). This may occur directly or via intermediary factors such as ZMYM2, which in turn might be recruited to PRC1.6-bound promoters through recognition of the SUMOylated complex. In both models, PRC1.6 acts as a transient repressor that primes chromatin for 5meC deposition. Notably, these are non-mutually exclusive mechanisms and may act in concert depending on developmental stage, target gene, or chromatin context.

An especially intriguing aspect of PRC1.6 is its association with H3K9 methylation machinery. Polycomb and H3K9me3 generally occur in exclusive regions of the genome: facultative and constitutive heterochromatin, respectively [70]. Given the general correlation between H3K9me2/3 and 5meC, the particular complex composition of PRC1.6 might help steer investigation towards a mechanism of DNA methylation recruitment (Fig 2a).

The G9A/GLP heterodimer is the main histone methyltransferase complex responsible for H3K9me1 and H3K9me2 deposition. These enzymes play an essential role during mammalian development, as their absence leads to embryonic lethality around E9.5 [71,72]. HP1 proteins, in turn, contain a chromodomain that binds to methylated H3K9 (H3K9me2 and H3K9me3) and play important roles in the spreading of these chromatin marks [73]. As mentioned above, various studies have reported a role of G9A/GLP-H3K9me2 in the repression of some PRC1.6 targets [32,42,68]. Moreover, *G9a* KO mouse embryos (analyzed at the post-implantation stage, when normally 5meC levels have been reestablished) exhibited ectopic expression of some germline genes [74,75]. Pertinently, this gain of expression is correlated with low promoter DNA methylation [75]. In another study, triple KO (TKO) of *Cbx1*, *3*, and *5*, encoding for HP1β, γ, and α, also led to upregulation of germline genes, including PRC1.6 targets, in ESCs [76].

Curiously, *Pcgf6* KO in mouse ESCs did not show significant alterations in H3K9me2 levels, indicating that G9A might be recruited to PRC1.6 targets by an independent means. Additionally, the HP1β/γ conditional double KO (dKO) was not associated with upregulation of targets bound by PCGF6 and RING1B, arguing against the necessity for classical H3K9 methylation-mediated silencing in ESCs [33]. Nevertheless, it should be noted that in ESCs, silencing can be maintained by Polycomb components.

Adding another layer of complexity, SETDB1/H3K9me3 is also linked with a subset of PRC1.6 targets [22,23,47]. *Max* KO ESCs show decreased levels of H3K9me3 and SETDB1 over the promoter region of select germline genes (*Ddx4*, *Dazl*, *Sycp3*) [47]. These and other MAX targets are also upregulated in *Setdb1* KD ESCs [47]. Even if SETDB1 is not frequently described as a PRC1.6 subunit from mass spectrometry studies, this histone modifier can be co-immunoprecipitated with the MAX factor [47]. Pertinently, upregulated genes shared between *Setdb1* KO and *Dnmt* TKO mouse ESCs are predominantly germline-specific [77]. SETDB1 activity at PRC1.6 targets appears particularly dependent on E2F6 binding: SETDB1 and H3K9me3, not G9A and H3K9me2, are enriched at E2F6-repressed germline genes, and this enrichment decreases in the absence of E2F6. Moreover, SETDB1 and E2F6 inactivation leads to upregulation of the same set of genes [23]. Interestingly, ATF7IP, a crucial partner of SETDB1, is found in the interactome of PCGF6 [34] and is one of the hits in an RNAi screen for germline gene regulators [68]. Very recently, Uranishi and colleagues have demonstrated a role for MGA in mouse ESCs in the recruitment of SETDB1 at PRC1.6 targets through its interaction with ATF7IP [78].

### L3MBTL2: Polycomb and/or H3K9 methylation factor?

A key PRC1.6 factor that has been implicated in both Polycomb and H3K9 methylation activities is L3MBTL2, although sometimes those data come into contradiction. L3MBTL2 is part of the Malignant Brain Tumor (MBT) protein family of transcriptional repressors that includes enzymes bearing from two to four MBT domains, able to recognize mono- and di-methylated lysines on histone tails [79]. *L3mbtl2* ablation in mice results in embryonic lethality correlated with ineffective gastrulation [32,39]. Concordantly, *L3mbtl2* KO ESCs show a growth defect and fail to differentiate properly [32]. This phenotype could be rescued only if all 4 MBT domains of L3BMTL2 and the atypical C2C2 zinc finger domain were present in the reintroduced protein. These domains are also important for the efficient binding with HDAC1, G9A, and RING1B, as tested by co-IP experiments [32]. Interestingly, while the MBT domains of L3MBTL2 are capable of recognizing methylated histone lysines (such as H3K4me1, H3K9me1/2, H3K27me1/2, and H4K20me1/2) [80], this binding activity appears to be dispensable for its core function, as mutations predicted to disrupt methyl-lysine binding did not impair the rescue of the colony growth defect [32]. Consistently, L3MBTL2 was shown to be capable of binding and compacting chromatin independently of methyl-lysine marks [81].

Reduction of L3MBTL2 in a human cell line (HEK293) caused upregulation of a set of genes that also exhibited decreased H2AK119ub levels [81]. Although this was not accompanied by a loss of E2F6 binding to the same targets, suggesting a specific role for L3MBTL2 in promoting H2AK119ub deposition downstream of PRC1.6 DNA binding [81]. However, this was not entirely confirmed by a subsequent study, in which H2AK119ub was found neither dependent on L3MBTL2 nor sufficient for repression of target genes [32]. Selected L3MBTL2-targeted germline genes (*Ddx4*, *Tex19*, *Piwil2*, *Stk31*, *Tcam1*) and PRC1 and PRC2 common targets (including *Sox17* and *Foxa2*) show L3MBTL2 binding and depend on it for their repression. In *L3mbtl2* KO ESCs, some of these genes exhibit a significant decrease in HDAC1, G9A, E2F6, and RING1B binding, accompanied by increased H3 and H4 acetylation, decreased H3K9me2 levels, but surprisingly no reduction of H2AK119ub [32]. These contradictory scenarios underscore the importance of being cautious in generalizing PRC1.6 component behavior, as in different contexts (i.e., different target genes and/or cell types), this could variably affect the chromatin landscape and transcriptional output.

### USP7: PRC1.6 stabilizer, 5meC deposition facilitator?

Two recently published CRISPR-KO screen studies, both using the germline gene *Dazl* as a reporter, uncovered new transcriptional regulators acting as repressors of germline genes [82,83]. Among the proteins that were further characterized by Al Adhami and colleagues, the deubiquitinating enzyme ubiquitin-specific protease 7 (USP7) (also discovered in the screen from Gupta and colleagues) acts as a potent inhibitor of germline genes and seems to be crucial in the initiation of their silencing. Interestingly, this factor has also been implicated in the stabilization of several subunits of PRC1.6, and this seems to be dependent on its catalytic activity [84]. The KO of this protein is also associated with lower 5meC levels at several germline gene promoters [82]. Furthermore, USP7 has previously been implicated in the maintenance of 5meC [85,86]; however, it remains unclear whether the hypomethylation observed at germline genes in its absence results from a direct role in 5meC retention or reflects an indirect function, potentially by facilitating PRC1.6-mediated targeting of 5meC through stabilization of the complex [82,84].

## PRC1.6 targeting requirements

Over the years, a number of studies have assessed the binding of various PRC1.6 components, most of which have been mentioned in this review. This section will highlight two extensive descriptions of PRC1.6 binding genome-wide [22,53]. Stielow and colleagues profiled MGA, L3MBTL2, E2F6, and PCGF6 in a human cell line (HEK293) and mouse ESCs, providing a map of PRC1.6 *bona fide* binding sites in both of these cell types. They also provided important insights into the different recruitment mechanisms of this complex. For example, MGA proved essential for PRC1.6 chromatin binding, and rescue experiments elucidated the role of its T-box and bHLH DNA binding domains as being indispensable only for a subset of PRC1.6 loci. Furthermore, L3MBTL2 and E2F6 contribute to differential promoter binding [23,53].

Notably, PCGF6, despite not playing a predominant role in the targeting of PRC1.6, is essential to recruit RING1B to PRC1.6 sites [33,53]. Interestingly, while E2F6-dependent PRC1.6 sites were enriched for the E2F binding motif, L3MBTL2-dependent ones showed a predominance of the bHLH E-box motif, suggesting a role for this factor in the stabilization of MAX/MGA at promoters bearing these sequences. In turn, L3MBTL2 binding at a subset of germline genes in mouse ESCs seemed to be independent of PCGF6 but dependent on MAX/MGA [33].

Mochizuki and colleagues built on these findings by assessing the impact of H2AK119ub and H3K9me3 in a naïve ESC to EpiLC *in vitro* differentiation system that recapitulates the exit of naïve pluripotency [87]. They found that SETDB1 plays a more significant role than G9A in establishing silencing at PRC1.6 target sites [22]. While PCGF6 is essential for silencing and H2AK119ub establishment in ESCs, it plays a less critical role than MGA in H3K9me3 formation, 5meC deposition, and gene repression in EpiLCs [22]. Once again, this suggests a parallel H3K9me3 recruitment mechanism, via MAX/MGA. This is in line with the recent results obtained by Uranishi and colleagues on SETDB1 recruitment via MGA-ATF7IP interaction [78]. Nevertheless, another study focusing on select PRC1.6 targets (*Tex101*, *Taf7l*, *Rhox13*, *Tcam1*, *Tdrkh*) in mouse ESCs showed that binding of RING1B, RYBP, PCGF6, L3MBTL2, and MAX at these sites is decreased in *Pcgf6*, *L3mbtl2*, and *Max* single KO ESCs [88]. This highlights an interdependency between different subunits of PRC1.6 for the binding of the complex at least at some targets.

## PRC1.6-5meC crosstalk

Investigating the interplay between PRC1.6 and 5meC is particularly relevant in the context of early mammalian development. Importantly, numerous studies discussed above presented proteomics approaches investigating PRC1.6 composition and interactions, and none of these recovered DNMTs as signficant hits [23,29,30,32,33,81]. These results discourage the hypothesis of a direct recruitment through PRC1.6-DNMT interaction—or if it does occur, it must be very transient in nature. As discussed, the available data raise the possibility that the function of PRC1.6, or specific PRC1.6 components, in the naïve state is to silence germline genes during a transient window when 5meC levels are low, and then recruit DNA methylation machinery, after which PRC1.6 becomes dispensable. We will describe specific factors that may play a role in the switch from Polycomb to 5meC.

### H3K9 methylation axis-dependence

As mentioned, H3K9 methylation and 5meC are highly correlated in mammalian genomes, and a number of studies have examined a mechanistic connection between the two chromatin marks. For example, the G9A/GLP heterodimer can contribute to gene silencing by both H3K9me2 and 5meC pathways independently [89,90]. Notably, catalytically inactive G9A/GLP mutants were sufficient to silence targets in serum-grown (high 5meC) ESCs, and this was correlated with restoration of 5meC compared to *G9a*/*Glp* constitutive KOs that exhibit 5meC hypomethylation at these genes [89]. Similarly, Dong and colleagues reported a significant decrease in 5meC at PRC1.6 targets, such as *Sycp1*, *Dazl*, *Brdt*, *Spo11*, and *Tuba3* [90]. In the same study, which was primarily focusing on repetitive elements, the authors also propose a mechanistic link between G9A and DNMT3A recruitment at these sites [90]. Concordant with these results, another study demonstrated that G9A is required for *de novo* 5meC deposition at certain PRC1.6-bound sites during the transition from undifferentiated ESCs to differentiated cells [91]. Mechanistically, these authors found a role for the ankyrin repeat region of G9A in the potential direct recruitment of *de novo* DNMTs [91].

SETDB1 is also required for 5meC homeostasis across various genomic regions, including some germline genes, in ESCs [92]. Recently, Taglini and colleagues revealed the importance of the N-terminal region of DNMT3B in its activity at H3K9me3-marked loci. Through *in vitro* experiments, they demonstrated that this region interacts with HP1α, suggesting it may facilitate DNMT3B recruitment to H3K9me3-positive heterochromatin [93]. This result elicits the tantalizing hypothesis that the other HP1 proteins β and γ, which have been described as PRC1.6 subunits, could play a similar role in bridging DNMT3B at PRC1.6 target genes (Fig 2a). Pertinently, genetic studies reveal DNMT3B as the primary DNA methyltransferase targeting germline genes in post-implantation embryos [3,8].

Finally, the DNA methylation maintenance cofactor, UHRF1, binds H3K9me2/3 through its Tandem Tudor and PHD domains [94,95]. A recent study has also demonstrated that UHRF1 can directly interact with both DNMT3A and DNMT3B to stimulate *de novo* 5meC activity [96], however, this function has not been formally demonstrated at germline genes.

### A potential role for SUMOylation

SUMOylation, the PTM by the small ubiquitin-like modifier (SUMO), serves as an important regulator of cell identity by targeting distinct substrates that differentiate pluripotent from somatic states [97,98]. A major function of SUMO in ESCs is stabilizing silencing complexes, notably including PRC1.6 [98]. Several subunits of PRC1.6 are heavily decorated by this PTM in ESCs—including L3MBTL2, MGA, and PCGF6—and SUMOylation is thought to act as a “glue” for the complex assembly and integrity [98]. This is also the case for several other chromatin modifiers that have been implicated in crosstalk with this complex, like proteins mediating H3K9me2/3, DNMTs, and PRC2 [97].

Readers of this PTM are characterized by the presence of SUMOylation-interacting motifs (SIMs). Zinc-finger MYM-type 2 (ZMYM2) contains a SIM and has been implicated in favoring 5meC gain during peri-implantation development at PRC1.6 targets [99] (Fig 2b). However, the means of recruitment of this factor at PRC1.6 sites and the mechanism by which ZMYM2 promotes 5meC deposition are still not completely understood. Graham-Paquin and colleagues propose SUMOylation of PRC1.6 as the prerequisite for ZMYM2 co-binding, but also mention possible recruitment through ATF7IP. While considering a possible means to stimulate 5meC deposition, they suggest a link with H3K4 demethylation, as this residue shows hypermethylation in *Zmym2* KO ESCs and embryoid bodies and precludes 5meC deposition [99].

### Indirect recruitment via H3K4 demethylation

DNA methylation is the default state in the mammalian genome, with the exceptions being the majority of CGI promoters. One protective mechanism for CGIs against 5meC is the presence of H3K4me2/3, which prevents DNMT3A/B from accessing chromatin [100,101] (Fig 2b). While PRC1.6 proteomic experiments do not recover DNMT enzymes as significant interactors, the H3K4 demethylase KDM5C is associated with PRC1.6 machinery and germline gene repression [34,102–104]. One plausible scenario is that PRC1.6 recruits KDM5C to create a chromatin environment amenable to 5meC establishment. To wit, *Kdm5c* KO mice show ectopic expression of germline genes in brain tissues, and this is associated with increased H3K4me3 and reduced 5meC at their promoter [103]. Moreover, *Kdm5C* was also recovered in both *Dazl* reporter screens [82,83]. However, at least for the germline genes *Dazl* and *Taf7l*, repression is independent of KDM5C catalytic activity [83], which would argue against the indirect recruitment model. Interestingly, a recent preprint showed KDM5C binds the CGI promoter of several germline genes, facilitating 5meC gain during naïve ESC to EpiLC differentiation at some of these loci [104]. Notably, many others gain 5meC despite the absence of KDM5C and an increase of H3K4 methylation (including *Dazl*), suggesting that the contribution of the catalytic activity of this demethylase can vary depending on the locus. Future work is necessary to dissect the precise role KDM5C plays in the context of germline gene regulation and to uncover the local chromatin or genomic features that enable compensatory mechanisms in its absence.

Curiously, the factor WDR5 is also a core PRC1.6 subunit, and to our knowledge, not present in other PRC1 complexes [30]. Conversely, WDR5 is a member of the WRAD set of proteins that are present in all H3K4 methyltransferase complexes [105]. Loss of WRAD components leads to a total loss of all types of H3K4 methylation in mouse ESCs [106]. One purely speculative hypothesis is that PRC1.6 coopts WDR5 not to carry out its silencing function, *per se*, but instead to antagonize H3K4 methylation machinery at PRC1.6 targets. This model, of course, would need functional validation.

## Conclusions and perspectives

PRC1.6 has emerged as a key regulator of germline gene repression during early mammalian embryogenesis, acting as an essential precursor to the establishment of stable silencing mediated by 5meC. During the critical peri-implantation window, PRC1.6 appears to initiate repression of germline gene promoters when 5meC levels are still low, subsequently facilitating targeting of the *de novo* DNMTs to these loci. While the exact mechanism of DNMT recruitment remains to be fully elucidated, multiple lines of evidence suggest both direct and indirect modes of interaction. We raised a number of possible pathways that might explain the switch from PRC1.6 to 5meC. Multiple mechanisms could exist at individual loci, and variations may exist across different loci. It is also worth noting that the MAX factor is well-described as sensitive to 5meC when present in the E-box motif [107,108]. This raises the possibility that once 5meC is established, the mark antagonizes PRC1.6 binding, thus completing the switch of silencing modes.

PRC1.6-targeted germline genes exhibit high sensitivity to DNA methylation loss, with their upregulation serving as a remarkably specific transcriptional signature of global 5meC depletion across diverse tissues and developmental stages [109]. Germline genes can be distinguished from other genetic loci sensitive to loss of DNA methylation machinery (e.g., imprints, certain classes of TEs) because this class is derepressed in PRC1.6 mutants in naïve ESCs, where global 5meC levels are very low. However, studying the function of PRC1.6 as a potential “recruiter” of 5meC through genetic means is inherently challenging: disruption of PRC1.6 in naïve, hypomethylated ESCs leads to upregulation of these genes, which can, in turn, influence 5meC deposition at their promoters. Disentangling the distinct contributions and functions of individual PRC1.6 components and their interactors in the silencing of these genes should be a key focus of future studies.

From an evolutionary perspective, Polycomb and H3K9 methylation pathways appear to represent ancestral mechanisms for silencing germline genes, conserved in organisms lacking DNA methylation [110–112]. In vertebrates, such as mammals, 5meC likely became integrated as a robust, heritable layer of repression, safeguarding somatic identity by stably repressing germline gene expression [113]. Incidentally, there is meager evidence for embryonic DNA methylation reprogramming in non-mammalian vertebrates [113–115]. PRC1.6 components are generally highly conserved, and the Drosophila PhoRC complex contains many homologs of PRC1.6 subunits (e.g., HP1β, L3MBLT2, and MGA) [88]. However, the adaptation of PRC1.6 to provide transient silencing for germline genes and recruit DNA methylation machinery is likely a mammalian innovation.

The necessity of this epigenetic layering is underscored by pathological contexts. Human cancers typically exhibit vastly misregulated DNA methylomes [2]. As the name indicates, cancer-testis antigens are germline genes that are expression-markers in a variety of cancers due to aberrant loss of promoter DNA methylation [116]. Moreover, dysregulation of PRC1.6 interactors or DNA methylation enzymes such as G9A, KDM5C, and DNMT3B is linked to aberrant germline gene activation in cancers and neurodevelopmental disorders [117]. Loss of PRC1.6 function, particularly MGA, disrupts chromatin repression and permits oncogenic transcription factor binding (e.g., MYC), further emphasizing its role as a tumor suppressor [118]. These results argue for a role of PRC1.6 outside of the embryonic developmental phase in pathological contexts, in which reversion to an embryonic-like state necessitates PRC1.6 for the silencing of its targets. It should be noted that this is partially speculative, and the link with PRC1.6 dysregulation and the global 5meC hypomethylated state in cancer warrants continued investigation.

Finally, although not the focus of the review, it is necessary to mention that the repressive function of PRC1.6 may extend beyond its well-characterized germline gene targets to include specific classes of proviruses [119] and TEs, particularly endogenous retroviruses (ERVs). Although this role is relatively understudied, emerging evidence suggests PRC1.6 may play a part in their silencing. For example, MERVL elements are upregulated in *Max* KD ESCs, linking PRC1.6 with their silencing [47]. This repression may involve cooperation with other chromatin regulators like SETDB1 and DNMTs. Notably, RIF1—an interactor of PRC1.6 [34,120]—has been shown to promote silencing at ERV loci by recruiting histone methyltransferases and favoring 5meC enrichment [121]. RIF1 also stabilizes PRC1.6 and facilitates its targeting to 2 Cell-like (2C-like) genes (Box 1) and retroelements, helping maintain the barrier between totipotency and pluripotency [120]. Compellingly, MERVLs play an integral role in regulating zygotic genome activation and its associated transcriptional program [122]. One could imagine a scenario where MERVLs take advantage of a brief window where DNA methylation levels recede, only to be silenced by PRC1.6 as the embryo transitions to pluripotency, and then finally stably silenced by 5meC in somatic cell types. It is perhaps not surprising that genetic screens ostensibly searching for regulators of germline genes uncovered regulators of the 2C-like program [82,83]. MERVLs represent a case study in TE exaptation by the host organism, and the interplay with PRC1.6 deserves further investigation.

In summary, PRC1.6 sits at the crossroads of different silencing pathways—Polycomb repression, H3K9 methylation, and 5meC—working together to turn off germline genes and some repetitive elements. Future studies clarifying the interface between PRC1.6 and DNA methyltransferases, the roles of accessory factors (e.g., USP7, ATF7IP, ZMYM2, KDM5C), and the influence of PTMs will be essential to fully understand this critical regulatory hub in development and disease.

**Box 1. *In cellula* modeling of different developmental stages**

**2C-like cells (2CLCs)**

Although mouse embryonic stem cells (ESCs) are pluripotent, 2CLCs spontaneously arise under serum culture conditions, comprising ~0.1%–0.5% of the population [123,124]. These cells share some transcriptional and epigenetic features with 2-cell stage blastomeres, representing a transient, totipotent-like state. 2CLCs exhibit distinct chromatin features: a reduction in global DNA methylation, increased levels of active histone marks, and higher chromatin mobility compared to typical ESCs. Interestingly, 2CLCs partially reactivate germline genes, such as *Dazl*, *Asz1*, and *Spz1*, reflecting shared regulatory features between early embryonic and germline transcriptional programs [125]. This overlap arises in part from the reduction of repressive mechanisms—notably 5meC and PRC1.6-mediated silencing—that normally maintain germline gene repression in ESCs [126].

**Naïve ESCs (nESCs)**

The epigenetic landscape of ESCs is highly influenced by culturing conditions [127]. Serum-free medium supplemented with two inhibitors—PD0325901 (MEK inhibitor) and CHIR99021 (GSK3 inhibitor), collectively known as 2i—promotes a more homogeneous population with characteristics of the naïve pluripotent state, including reduced global 5meC levels (~30%). Aligning to the global landscape, germline gene promoters^(1)^ generally show a low 5meC enrichment in nESCs [22]. *Pcgf6* is well-expressed in these conditions, which matches the *in vivo* pattern [33,128]. As discussed above, there is several evidence for the repressive activity of PRC1.6 towards a subset of germline genes in nESCs.

**Primed ESCs (pESCs)**

ESCs maintained in serum-containing medium typically exhibit approximately 70%–80% global CpG methylation. These conditions give rise to a heterogeneous population, comprising cells at various stages of pluripotency (for example, 2CLC). In serum-grown ESCs, germline gene promoters^(1)^ are characterized by intermediate to high 5meC levels [22]. *Pcgf6* expression is similar to nESCs [33], and as seen in this review, the repressive role of PRC1.6 towards a subset of germline genes is well-established in these cells.

**Epiblast-like cells (EpiLCs)**

To better model the primed state of the epiblast, an alternative to serum involves differentiating nESCs in the presence of FGF2 and Activin A, generating epiblast-like cells (EpiLCs). These cells recapitulate many of the transcriptional and epigenetic features of the E5.5–E6.5 post-implantation epiblast stem cells. After two days of differentiation in EpiLC media, the global 5meC levels are already at 70%–80%, the same as for extended EpiLCs (more than two days of differentiation). EpiLCs at day 2 show intermediate-to-high 5meC enrichment at germline gene promoters^(1)^. The subset bound by PRC1.6 generally acquires the mark gradually, reaching higher levels in extended EpiLCs [22].

**Primordial germ cell-like cells (PGCLCs)**

EpiLCs can be further differentiated into primordial germ cell-like cells (PGCLCs) using a defined cytokine cocktail (typically including BMPs, LIF, SCF, and EGF), which exhibit a transcriptional program reminiscent of *in vivo* PGCs (E7.25–E13.5) [129,130]. This is marked by the reactivation of germline genes and the repression of somatic programs. Depending on culturing conditions and timing, PGCLCs can present a highly similar 5meC landscape to PGCs, generally characterized by global hypomethylation [131]. In these cells, PRC1.6 repressive activity towards germline genes seems to be alleviated through several means [69,132].

^(1)^Information on germline genes refers to the subset that gains dense promoter 5meC by E6.5 and upregulation in *Dnmt3a*/*3b* DKO at E8.5 embryonic stage *in vivo*, defined as DNA-methylation sensitive germline genes [3]. Importantly, there exist locus-specific exceptions to the reported trends.
